# Accurate Dereplication of Bioactive Secondary Metabolites from Marine-Derived Fungi by UHPLC-DAD-QTOFMS and a MS/HRMS Library

**DOI:** 10.3390/md12063681

**Published:** 2014-06-20

**Authors:** Sara Kildgaard, Maria Mansson, Ina Dosen, Andreas Klitgaard, Jens C. Frisvad, Thomas O. Larsen, Kristian F. Nielsen

**Affiliations:** Department of Systems Biology, Technical University of Denmark, Soeltofts Plads 221, Kgs. Lyngby DK-2800, Denmark; E-Mails: sarki@bio.dtu.dk (S.K.); maj@bio.dtu.dk (M.M.); idos@bio.dtu.dk (I.D.); ankl@bio.dtu.dk (A.K.); jcf@bio.dtu.dk (J.C.F.); tol@bio.dtu.dk (T.O.L.)

**Keywords:** marine-derived, MS/MS, dereplication, library, peptaibiotics, metabolomics

## Abstract

In drug discovery, reliable and fast dereplication of known compounds is essential for identification of novel bioactive compounds. Here, we show an integrated approach using ultra-high performance liquid chromatography-diode array detection-quadrupole time of flight mass spectrometry (UHPLC-DAD-QTOFMS) providing both accurate mass full-scan mass spectrometry (MS) and tandem high resolution MS (MS/HRMS) data. The methodology was demonstrated on compounds from bioactive marine-derived strains of *Aspergillus*, *Penicillium*, and *Emericellopsis*, including small polyketides, non-ribosomal peptides, terpenes, and meroterpenoids. The MS/HRMS data were then searched against an in-house MS/HRMS library of ~1300 compounds for unambiguous identification. The full scan MS data was used for dereplication of compounds not in the MS/HRMS library, combined with ultraviolet/visual (UV/Vis) and MS/HRMS data for faster exclusion of database search results. This led to the identification of four novel isomers of the known anticancer compound, asperphenamate. Except for very low intensity peaks, no false negatives were found using the MS/HRMS approach, which proved to be robust against poor data quality caused by system overload or loss of lock-mass. Only for small polyketides, like patulin, were both retention time and UV/Vis spectra necessary for unambiguous identification. For the ophiobolin family with many structurally similar analogues partly co-eluting, the peaks could be assigned correctly by combining MS/HRMS data and *m*/*z* of the [M + Na]^+^ ions.

## 1. Introduction

Due to the cosmopolitan occurrence of many bioactive compounds, most natural product extracts contain compounds that have previously been characterized, despite intelligent selection of new organisms. This is of particular importance in primary screens where the target is usually non-selective, which inevitably leads to a high rediscovery rate of generally toxic compounds [1,2]. 

Microorganisms from the marine environment are a promising source of new bioactive compounds based on new chemical scaffolds [3,4,5], with the majority of known compounds originating from bacterial species such as *Salinospora* [6], *Pseudoalteromonas* [7,8], and *Vibrio* [9]. However, the subject of marine fungi is of much debate as most marine isolates have been found in mangrove and intertidal zones [4,10,11], rather than in true marine habitats; thus, no strict definition of “true marine fungi” currently exists [12]. Nonetheless, marine-derived fungal strains have yielded a plethora of biologically active compounds [5,13], with isolates of *Penicillium* and *Aspergillus* as the most common sources. These have mainly been isolated from substrates such as driftwood [14] and macroalgae [15], but also in deep-sediments [3,16,17]. *Aspergillus sydowii* is probably the most well-known example, identified as the cause of sea fan disease [18], but also the source of bioactive compounds [19]. It remains obscure whether these represent true marine isolates or just opportunistic strains that have adapted to the marine conditions [12]. From a drug discovery perspective, this might be of less importance, if the opportunistic strains produce different bioactive compounds than their terrestrial counterparts. 

Several approaches to the dereplication process exist; for fast screening of extracts the aggressive dereplication approach can be very efficient [20]. This approach is based on accurate mass, isotopic patterns, and preferably selective adducts used for large batch searches of possible metabolites (up to 3000 compounds), e.g., based on all compounds described by a single genus. Yet, it returns false positives that need to be sorted away. The approach is currently not suited for organisms with limited taxonomic information. False positives can be circumvented by adding tandem MS with accurate mass determination of fragment ions (MS/HRMS) which can be automatically co-acquired using auto-MS/HRMS experiments (data-dependent acquisition of MS/HRMS spectra) [21]. This can now be achieved on both time-of-fight (TOFMS) and fourier transform (FTMS) mass spectrometers as well as Orbitrap and Q-Exactive instruments [22,23,24,25]. To achieve high quality MS/MS spectra, Agilent Technologies have chosen to acquire spectra at three different fragmentation energies, 10, 20 and 40 eV, as this often provides significant higher quality than e.g., a ramped spectrum from 10 to 40 eV [26]. The acquired MS/HRMS data can then be matches with the possible candidates using *in silico* fragmentation tools that can sort out poor matches [27,28].

For fast tentative identification of natural products, an automatic MS/HRMS spectral library search would be very efficient, if suitable natural products libraries existed. However, Massbank [29] and Metlin metabolomics library [30] (~10,000 compounds with spectra) only contain few microbial natural products. The current status will persist until it is required to publish MS/MS data with novel structures, for which there are now public depositories such as MetLin, Massbank and/or Global Natural Products Social Molecular Networking (GnPS) [31] in the making at time of writing). Nevertheless, a major barrier is that MS/MS spectra of small molecules are inconsistent between instruments, in particular between ion-trap and collision cell-based instruments [32]. Also, compared to fragmentation of linear peptides [33] and lipids [34], fragmentation of natural products are much less predictable, since they often contain more condensed and highly complex ring systems: In consequence *in silico* predictors cannot predict a fragmentation spectrum, but to some extent, verify some fragments from a structure in a spectrum [27,28].

For smaller natural products libraries, different algorithms have been used to search MS/MS spectra for the tentative identification (absolute identification always requires a nuclear magnetic resonance (NMR) validated reference standard). Fredenhagen *et al.* [35] searched low resolution MS/MS data with the National Institute of Standards and Technology (NIST) algorithm developed for full scan EI^+^ spectra and the Mass Frontier software for MS*^n^* spectra and found the latter to be superior. El-Elimat *et al.* [2] used ACD-IntelliXtract that also includes accurate mass of the fragments, but does not use the parent ion data as search entry. A comprehensive review on algorithms can be found in Hufsky *et al.* [28]. Recently, a networking MS/MS strategy has been published from the Dorrestein/Bandeira labs [36,37], where MS/MS spectra are compared pairwise to yield clusters of structurally related compounds. However, back integration/deconvolution of raw data to find corresponding full scan data and linking MS/MS spectra of adducts belonging to the same molecular feature as well as retention time still needs to be done manually and is thus very time consuming. 

In this current study, we demonstrate the use of our MS/HRMS library search to dereplicate known compounds in bioactive extracts from marine-derived *Aspergillus*, *Penicillium*, and *Emericellopsis* strains. Extracts were selected from a screening conducted as a part of the PharmaSea project [38]. 

Ultra-high performance liquid chromatography-diode array detection-quadrupole time of flight mass spectrometry (UHPLC-DAD-HRMS) with auto- tandem high resolution mass spectrometry (MS/HRMS) analysis was used to screen the extracts and subsequently, MS/HRMS data was matched against a newly constructed library of 1300 compounds (10, 20, and 40 eV spectra) using the Agilent search algorithm. This algorithm is an integral part of the Agilent MassHunter software, which can subtract background and merge spectra over a chromatographic peak into a single spectrum prior to automatic search against the library. To assess the limitations and inherent bias of the library, we compare the results with the aggressive dereplication approach [20] based on accurate mass, isotope pattern, and lists of taxonomically relevant compounds. Specificity is tested on a number of small polar analytes, showing the importance of including retention time and appropriate search parameters for compounds with less characteristic spectra. Finally, comparison with UV/Vis detection was done for a number of poorly ionizing compounds showing the value of this additional cheap detector. 

## 2. Results and Discussion

**Figure 1:**
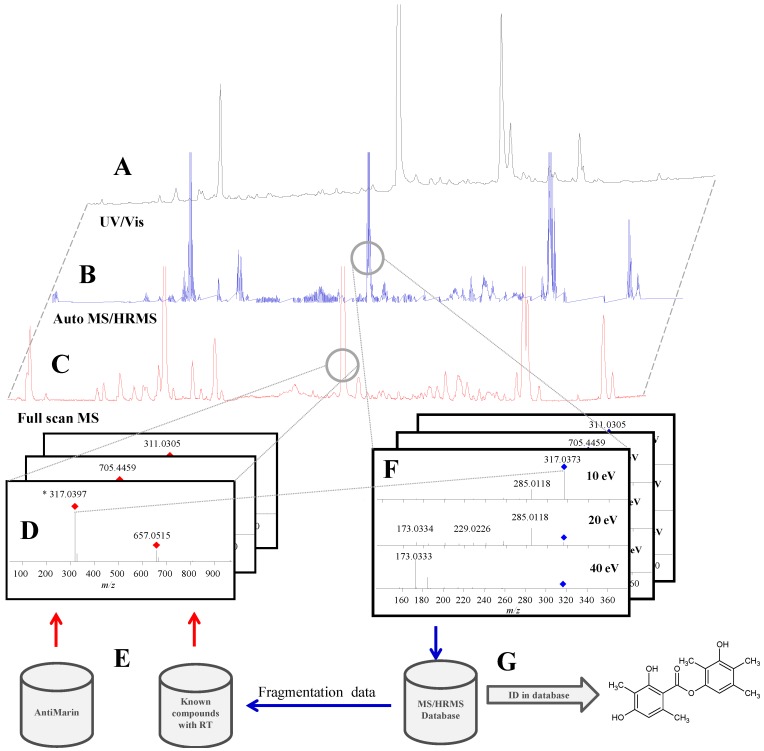
Overview of the screening setup where ultra-high performance liquid chromatography (UHPLC) with three detection methods is used. (**A**) ultraviolet/visual (UV/Vis) for poorly ionizing compounds; (**C**,**D**) full scan high resolution mass spectrometry (HRMS) screening; (**B**,**F**) MS/HRMS identification using the MS/HRMS library (**G**). Elemental compositions from compounds known from literature and previous studies were searched for in the full scan data (**E**,**D**).

Figure 1 illustrates the overall screening concept used in this study, where UHPLC-DAD-QTOF data are analyzed in three different ways: (i) MS/HRMS data searched directly in MS/HRMS library; (ii) aggressive dereplication of the full scan HRMS data using search lists of known compounds; (iii) UV/Vis detection for poorly ionizing compounds. Finally, an unbiased peak-picking algorithm was used to highlight completely novel compounds. For dereplication of previously described compounds and novel isomers, all four approaches were combined as illustrated in the examples of *Penicillium bialowiezense* (Section 2.2.1) and *Aspergillus insuetus* (Section 2.2.2). Specificity problems with MS/HRMS searching are illustrated for patulin and compounds with the same elemental composition (Section 2.1.5).

### 2.1. Data Acquisition and Library Creation

#### 2.1.1. Chromatographic Separation

The gradient was developed to provide the highest peak capacity in extracts from *Aspergillus niger* and *A. nidulans* with emphasis on not losing polar alkaloids (e.g., pyranonigrins and nigragillins) and small organic acids. This led to the use of the more polar phenyl-hexyl Poroshell column (compared to C_18_) as well as a low start of the gradient (10% acetonitrile). This retains highly polar compounds such as patulin and type B trichothecenes slightly better than C_18_. However, the long column required a longer gradient and equilibration time leading to half the productivity, but better opportunities for more MS/MS experiments. The high temperature of 60 °C was needed in order to keep the back pressure below the limit of the 2.7 µm Poroshell column. The method yielded an excellent peak distribution and narrow peak width compared to other methods [2], which allowed for higher quality spectra of most compounds in an extract. Injection volume had to be kept low (1 µL) to avoid peak broadening of polar peaks as samples were dissolved in methanol. However, in some projects less had to be injected (as little as 0.1 µL) as strongly ionizing compounds in high concentration resulted in broad peaks due to peak broadening in the ion-source which was further enhanced by the limited linearity of the time of flight (TOF) detector.

#### 2.1.2. Mass Accuracy and Isotopic Ratio

**Figure 2:**
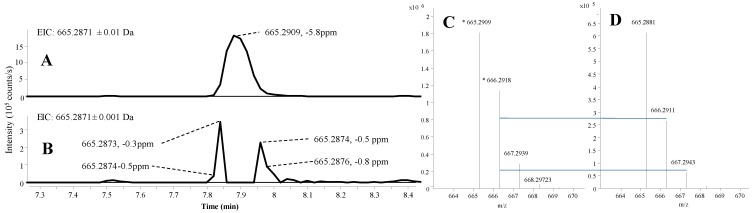
Ultra high performance liquid chromatography-electrospray ionization extracted ion chromatograms (UHPLC-ESI^+^ EIC) of asperazine [M + H]^+^ in an extract from *Aspergillus tubingensis*, showing the excellent mass accuracy until saturation in the peak apex. (**A**) EIC at ±0.01 Da; (**B**) EIC at ±0.001 Da; (**C**) spectrum at peak apex; and (**D**) spectrum at a non-saturated part of the peak. The vertical lines between **C** and **D** indicate the theoretical isotopic abundance of the A + 1 and A + 2 isotopomers.

Currently, time of flight mass spectrometry (TOFMS) and fourier transform mass spectrometry (FTMS) instruments provide similar mass accuracy when using a lock mass, but the TOFMS instruments still have problems with detector overload [39,40] as illustrated in Figure 2, where the mass accuracy and isotope ratio is compared between overloaded and non-overloaded parts of a chromatographic peak. As high intensity peaks are unavoidable, MassHunter was set to handle this by using only non-overloaded MS scans from the front and end of the chromatographic peaks during the peak picking and integration, similar to other TOFMS manufacturers like Waters. Currently, this cannot be handled by any third-party software like ACD-IntelliXtract or open source software like XCMS and MZmine.

On the up-side, quadruple time of flight mass spectrometry (QTOFMS) instruments have a much higher scan frequency of both full scan and MS/HRMS scans without losing resolution as is the case on the FTMS instruments (resolution proportional to scan time). When not using overloaded ion clusters (Figure 2) our data provided isotopic ratios <±2% relative to the theoretical distribution as also observed elsewhere [20] while for Orbitrap data it might be as much as ±35% [24]. Since an accurate isotope ratio is the most efficient way to differentiate candidate elemental compositions within the instrument accuracy [41], the QTOFMS instruments are superior to the FTMS instruments in this point.

In some samples, high intensity peaks suppressed the lock mass ions in certain scans, resulting in up to 100 ppm mass error in cases where the instrument had not been tuned and calibrated for several days. Since MassHunter cannot automatically find scans with intact lock mass in other places in the data file, one needs to be aware of this problem to manually correct it if needed. Here, the MS/HRMS library still identified the correct compounds, underlining how this approach is very robust against mass errors from over-loaded peaks.

#### 2.1.3. Precursor Selection

A major challenge when using liquid chromatography-mass spectrometry (LC-MS)/MS libraries is the reproducibility of fragmentation patterns between different instruments and different instrument manufactures [42]. A major goal for the establishment of this library was to minimize variability due to changes in mobile phase composition and ion-source settings. This was done by including not only MS/HRMS from [M + H]^+^, but also from the other predominant pseudo molecular ions such as [M + Na]^+^ and [M + NH_4_]^+^ (for intensities >50% of [M + H]^+^). Likewise, were MS/HRMS spectra of [M + H − (H_2_O)_n_]^+^, [M + H − HCOOH]^+^, and [M + H − CH_3_COOH]^+^ ions included when the full scan signal(s) were more intense than [M + H]^+^, similar to Fredenshagen *et al.* [35]. When fragmentation of [M + Na]^+^ only resulted in the loss of Na^+^ to give the neutral molecule, the search algorithm gave false positives from any ion at the right *m*/*z*. MS/HRMS data from the stable [M + Na]^+^ was therefore only included when resulting in specific fragments (~50% of the cases). Still, *m*/*z* of [M + Na]^+^ is important for correct mass assignment of fragile molecules, where [M + H]^+^ is not present due to spontaneous losses. Furthermore, in cases where in-source fragmentation of Compound A coincidentally results in production of Compound B also present in the library, the *m*/*z* of [M + Na]^+^ can assist in correct assignment, as demonstrated. 

In the negative ionization mode, [M − H]^−^ is most often the dominant ion detected [43] while the formation of [M − HCOO]^−^ (if formate is used as a buffer) seems to be very interface dependent [20], but very important for molecules not containing any acidic protons, and it was included when more than 50% of [M − H]^−^ occurred. This resulted in the detection of highly active compounds like Type A and C trichothecenes, patulin, and aphidicolins not detected in other studies [2,35].

Part of the library (277 compounds) is available in PCDL format for download from the homepage of the Technical University of Denmark [44].

#### 2.1.4. Fragmentation

In order to compensate for the high variation in energy needed to fragment natural products, the library was based on three distinct fragmentation energies (10, 20, and 40 eV) unlike existing microbial MS/MS libraries that are based on a single, fixed energy [2,35]. The high energy of 40 eV is needed to fragment larger, more stable molecules, while 10 and 20 eV are more gentle settings for smaller, more fragile molecules. This combination of energies also meant that the forensic science [26] and the Metlin libraries [35] which are also available for the MassHunter could be directly used. The latter, in particular, contains many lipids, prostaglandins, intracellular primary metabolites, small aromatics, amino acids, vitamins, *etc.*, which are also produced by fungi. The only cases where insufficient fragmentation was observed for all three energies were fusigen, SMTP-7 and 8, where only low intensity losses of formate and one other ion were observed in ESI^+^. Thus, projects analyzing compounds with masses above 1000 Da should include additional fragmentation energies of e.g., 60–80 eV, as large single charged molecules are less disposed to fragment on the collisions with N_2_. This is mainly due to simple energy kinetics (*E*_kin_ = ½ × *m*/*z* × *v*^2^) where the ion-velocity in the collision cell is proportional to the square root of the mass.

Small (<200 Da) aromatic acids, pyrones, and lactones will statistically have less specific fragmentation reactions, which is observed in practice as loss of H_2_O, HCOOH, and CO_2_ [43]. Combined with an increase in the number of natural products with the same mass with decreasing mass (down to 220 Da) there is a double bias towards poor specificity of MS/MS of low mass compounds [43].

#### 2.1.5. Library Scoring

Searching MS/HRMS spectra against the MS/HRMS library in MassHunter allows for three types of scorings: (i) using the parent mass and forward scoring that matches peaks in the unknown spectrum against the library spectrum; (ii) using parent mass and reverse scoring that matches peaks in the library spectrum against the unknown spectrum [28]; (iii) using reverse scoring but not the parent mass, called similarity, for finding compounds sharing fragment ions but having different molecular masses. 

**Figure 3:**
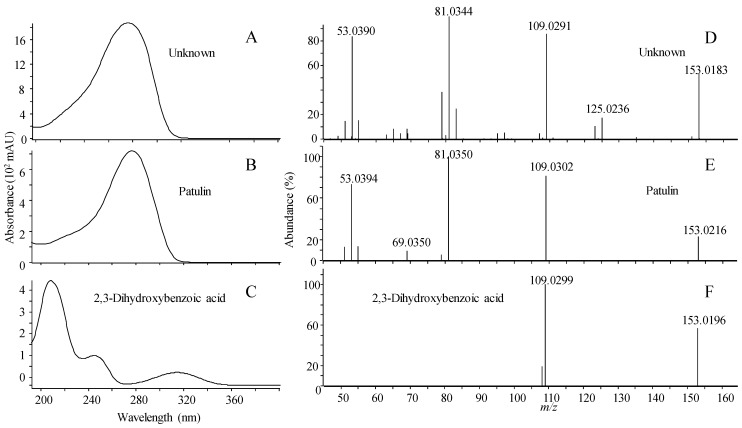
UV/Vis spectrum (**A**) and MS/HRMS spectrum at 10 eV (ESI^−^); (**D**) of unknown peak identified as patulin, compared to the patulin reference standard (**B**,**E**); and 2,3-dihydroxybenzoic acid (**C**,**F**). Identity was confirmed by correct retention time.

The pitfalls of scoring can be illustrated with patulin, a bioactive “nuisance” compound that is widely distributed in fungi that cause interference in many types of bioassays [45,46,47]. Patulin was identified in ESI^−^ in marine-derived strains of *Penicillium antarcticum* (Figure 3). Patulin shares the same elemental composition (C_7_H_6_O_4_) with six other compounds included in the library (Table 1), which all to a certain extent exhibited similar fragmentation patterns under the same CID condition. Using reverse and forward scoring, all library spectra belonging to compounds with the same elemental composition are in the matching pool. For reverse scoring there is an increased risk of wrong compound identification compared to forward scoring as the search algorithm in this case only looks for peaks present in the library spectra, disregarding peaks present in the unknown spectrum that are not present in the library spectra.

As seen in Figure 3, patulin and 2,3-dihydroxybenzoic acid had a similar ratio of the *m*/*z* 109.0287 fragment ion corresponding to the loss of CO_2_ (CID 10 eV). 2,3-dihydroxybenzoic acid does not show any additional peaks in the 10 eV spectrum while patulin produces several. Reverse scoring only matched the two shared peaks in the unknown spectrum, resulting in 2,3-dihydroxybenzoic acid as the best match, while forward scoring, where all peaks in the spectrum are matched with the library spectrum, yielded patulin as the best match (Figure 3). The identification was verified by an authentic standard of patulin matching full scan MS, MS/HRMS, retention time and the UV spectrum where the slow slope from 200 to 240 nm prior to the main absorption at 276 nm. Deconvolution of all ions in the patulin full scan spectrum showed that it was not a false positive detection due to two or more co-eluting compounds.

**Table 1:** Comparison of MS/HRMS spectra of all C_7_H_6_O_4_ compounds in the MS/HRMS database against each other using forward and reverse scoring.

Name	RT (min)	Compound	Forward/Reverse Scoring (%)						
			1	2	3	4	5	6	7
Patulin	3.15	1	100	28/50	20/62	27/65	29/90	28/87	25/52
2,3-dihydroxybenzoic acid	3.85	2	28/32	100	60/68	63/71	97/90	76/86	0/0
2,4-dihydroxybenzoic acid	3.74	3	20/29	60/86	100	86/86	55/78	88/88	6/14
2,6-dihydroxybenzoic acid	3.87	4	21/29	63/86	86/98	100	58/79	80/92	6/14
3,4-dihydroxybenzoic acid	2.80	5	29/33	97/97	55/61	58/64	100	78/87	0/0
3,5-dihydroxybenzoic acid	2.63	6	29/33	97/97	55/61	58/64	78/87	100	0/0
Terreic acid	3.99	7	25/44	0/0	6/39	6/38	0/0	0/0	100

Inevitably, an unknown spectrum will contain more noise from co-eluting compounds compared to the library spectrum, another reason why reverse scoring is also valuable. This underlines the importance of using both forward and reverse scoring when evaluating matches from library searches in order to get the correct identification, thus multiple search types are recommended (e.g., using a minimal score of 50% for forward and 70% for reverse). It is further demonstrated that there is a need for orthogonal data like UV/Vis for dereplication of certain compound classes.

### 2.2. Dereplication of Marine-Derived Fungi

Fifteen marine-derived strains from different species belonging to *Penicillium*, *Aspergillus*, and *Emericellopsis* were fractionated and screened for their anti-microbial [48], anti-inflammatory [49], central nervous system (CNS) [50], and anticancer activity (unpublished assay based on glioblastoma stem cells), that resulted in 35 active fractions to be evaluated for their chemistry. Here, we present three of those as cases to illustrate the advantages and challenges using a MS/HRMS library for screening and dereplicating active fractions during a screening campaign. Analyzing the data file for MS/HRMS data, including peak picking, integration, and the final matching against 1300 compounds took 30–60 s on a standard laptop, thus providing a fast and easy first examination of active fractions. 

#### 2.2.1. Active Components from a Marine-Derived *Penicillium bialowiezense* Strain

The extract of a *Penicillium bialowiezense* strain (IBT 28294) from a North Sea water sample displayed activity in a CNS assay [51] and anticancer assay (unpublished assay). *P. bialowiezense* is closely related to *P. brevicompactum,* and they are morphologically, genetically, and chemically very difficult to differentiate [52]. They are cosmopolitan species found across an amazing number of habitats such as seaweed, humid indoor environments, soil, and various vegetables and fruits [52,53]. Thus the marine-derived isolate used in this study is likely an opportunist in the marine environment, making the exclusion of known compounds even more important.

**Figure 4:**
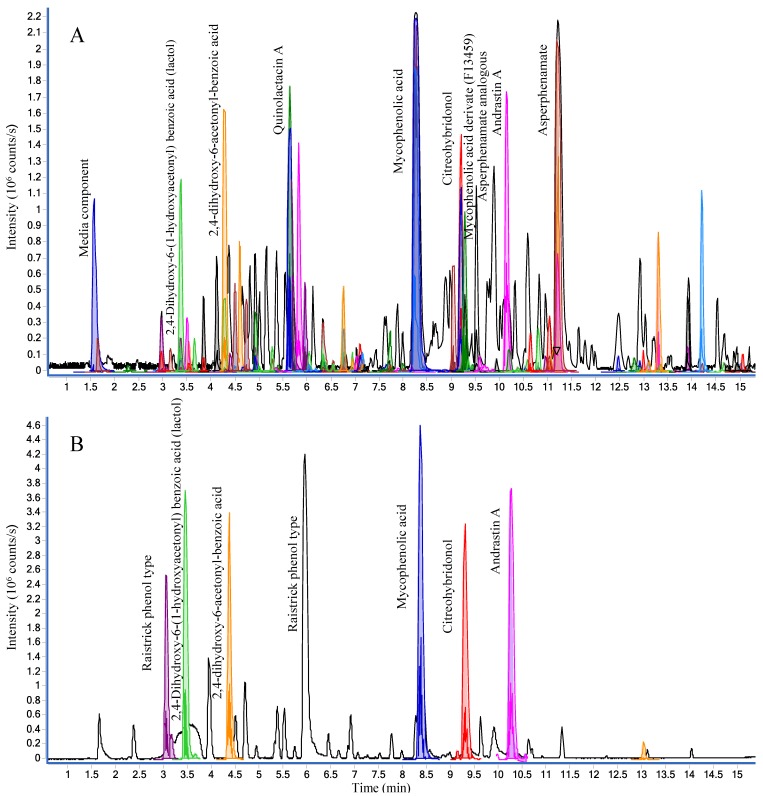
Base peak chromatograms (BPC) of the crude extract of *P. bialowiezense* in both positive (**A**) and negative (**B**) ESI modes. Peaks of compounds identified by MS/HRMS using forward scoring are colored.

The crude extract analyzed in both positive and negative ionization mode can be seen in Figure 4 with the tentative identification of all major peaks: mycophenolic acid, mycophenolic acid derivate (F13459), asperphenamates, andrastin A, quinolactacin A, citreohybridonol, and raistrick phenols [54], all of which have previously been reported from terrestrial fungi [55].

**Figure 5:**
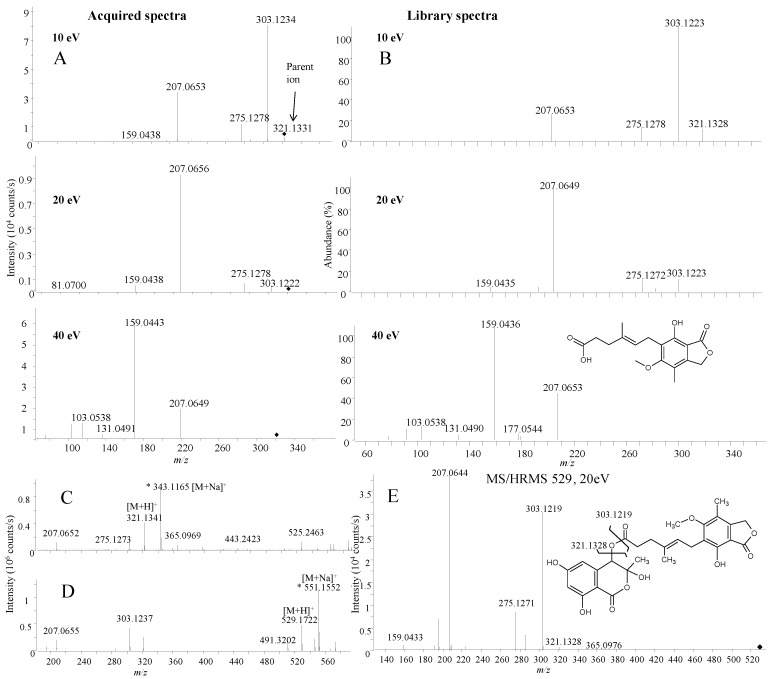
MS/HRMS spectra (*m*/*z* 321) for mycophenolic acid in the active fraction (**A**) compared to library spectra (**B**) at 10, 20 and 40 eV; (**C**) full scan spectrum of mycophenolic acid showing a [M + Na]^+^ ion at *m*/*z* 343; (**D**) full scan spectrum of F13459 showing a [M + Na]^+^ ion at *m*/*z* 551; (**E**) MS/HRMS at 20 eV for [M + H]^+^ of F13459 including structure of the compound.

The active fractions were found to contain mycophenolic acid (Figure 4 and Figure 5), which is the active compound in the prodrug CellCept^®^ (Mycophenalate mofetil) used as immunosuppressant in transplant medicine [56]. Several other activities have been reported including antiviral, antitumor [57], and CNS [56,58], in line with the activity observed in this study. The extracted MS/HRMS spectra (Figure 5A) in ESI^+^ were compared to the mycophenolic acid standard in the MS/HRMS library (Figure 5B) with high scores (>90%) using both reverse and forward searching based on the accuracy of the parent ion (−0.31 ppm for [M + H]^+^ 321.1328) and specific and abundant fragment ions at *m*/*z* 207.0649 [C_11_H_11_O_4_]^+^ and 159.0436 [C_10_H_7_O_2_]^+^.

In addition, with ESI^+^ reverse and forward scoring, a second compound was detected as mycophenolic acid itself but at a wrong retention time and not producing a [M + Na]^+^ ion (Figure 5D), indicating that it was a fragment from a larger molecule. HRMS of the [M + H]^+^ at *m*/*z* 529.1722 (Figure 5D) was used to tentatively identify the compound as a mycophenolic acid derivate F13459 previously isolated as a racemate from *Penicillium* sp. [59,60]. 

The identity was verified by MS/HRMS fragmentation of *m*/*z* 529.1722 into the same ions observed from MS/HRMS of [M + H]^+^ for mycophenolic acid (Figure 5E). F13459 might act as a natural prodrug that by hydrolysis loses the isocoumarin portion, leaving the active compound, mycophenolic acid (Figure 5E). The lost portion corresponds to the lactol form of the raistrick phenol, 2,4-dihydroxy-6-(1-hydroxyacetonyl) benzoic acid(Figure 5E) that was also detected in the extract (Figure 4).

**Figure 6:**
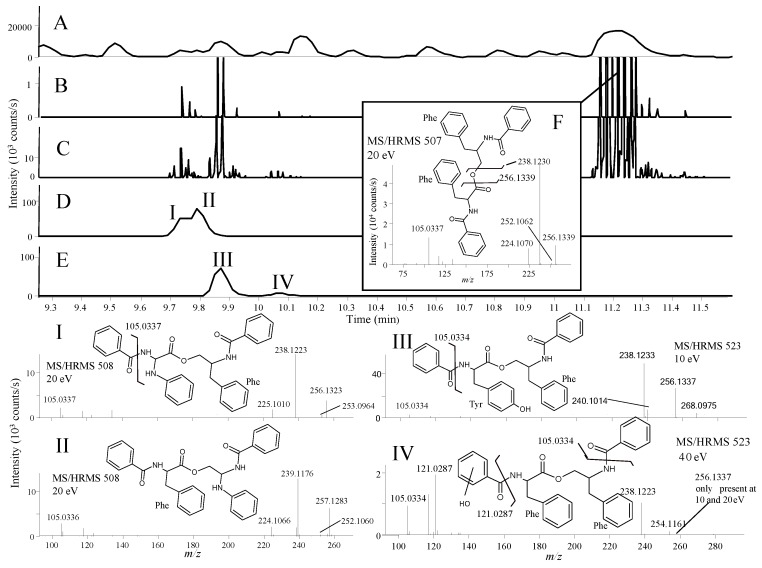
BPC chromatogram of the crude *P. bialowiezense* extract (**A**); EIC from MS/HRMS showing fragment ions (**B**) *m*/*z* 256.1333 and (**C**) 238.123; EIC full scan showing (**D**) *m*/*z* 508.2232 ± 0.005 and (**E**) 523.2211 ± 0.005; (**F**) MS/HRMS spectrum at 20 eV of asperphenemate. (**I**) to (**IV**) show the tentatively assigned isomers of asperphenamate and their positions in the chromatogram.

In the fraction displaying anticancer activity (unpublished assay), the library analysis led to the tentative identification of the fungal anticancer metabolite, asperphenamate (Figure 6F) [61]. A group of peaks eluting close to asperphenamate shared their major fragment ions as found using similarity searching (parent ion not used), which showed the presence of four novel asperphenamate analogues with the tentative structures I to IV (Figure 6). Unambiguous structure verification of course requires isolation and elucidation using nuclear magnetic resonance (NMR) spectroscopy.

Asperhenamate and three of the analogues (I, III, and IV) shared dominant fragment ions at *m*/*z* 238.1230 and 256.1339 (Figure 6B,C), corresponding to [C_16_H_18_NO_2_]^+^ and [C_16_H_16_NO]^+^ formed from the right side of the molecule by cleavage of the ester-bond followed by water loss. The most abundant asperphenamate analogue (III) had a [M + H]^+^ with *m*/*z* 523.2211, corresponding to an addition of an oxygen atom. This indicated replacement of the phenylalanine by a tyrosine in the asperphenamate skeleton, corroborated by the fragment ions at *m*/*z* 268.0975 [C_16_H_14_NO_3_]^+^ and 240.1014 [C_15_H_14_NO_2_]^+^ (Figure 6III) as opposed to 252.1062 [C_16_H_14_NO_2_]^+^ and 224.1070 [C_15_H_14_NO]^+^ in asperhenamate (Figure 6F). These fragments matched the left side of the molecule formed from the ester cleavage followed by the loss of CO. The fragment 105.0334 [C_7_H_5_O]^+^ corresponding to the benzoyl part was present in both asperphenamate and the analogues, and the lack of an ion at *m*/*z* 121.0287 also supported the presence of the tyrosine (Figure 6III).

The other analogue IV with the same accurate mass as III had a similar fragmentation pattern with addition of the most prominent fragment ion 40 eV at *m*/*z* 121.0287 [C_7_H_5_O_2_]^+^. This fragment could match the presence of an extra oxygen atom in the benzoyl part instead of the phenylalanine part. The last two analogues (I and II) had [M + H]^+^ *m*/*z* 508.2232 with similar fragmentation patterns to asperphenamate. Analogue II had *m*/*z* 239.1176 [C_15_H_17_N_2_O_2_]^+^ and *m*/*z* 257.1283 [C_15_H_15_N_2_O]^+^ as major fragment ions not present in the asperphenamate MS/HRMS spectrum (Figure 6F), showing a replacement of a CH with an N atom, presumably in the phenylalanine moiety to the right of the ester bond, as a fragment ion corresponding to change in the benzoyl part was not observed. For Analogue I, the two ions differentiating it from asperphenemate were *m/z* 253.0964 [C_15_H_13_N_2_O_2_]^+^ and 225.1010 [C_14_H_13_N_2_O]^+^ (Figure 6I), which also corresponded to the replacement of a CH with a nitrogen atom, in this case to the left of the ester bond (Figure 6I) and, as in the example with II, showed a lack of extra fragments.

**Figure 7:**
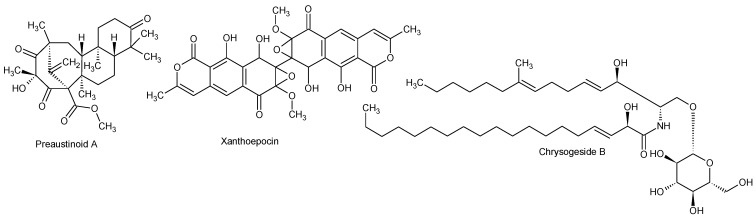
Structures of preaustinoid A, xanthoepocin, and chrysogeside B.

As the MS/HRMS library only covers about 5% of the compounds reported from fungi in AntiMarine (2012), though with a higher coverage of *Pencilllium* and *Aspergillus* compounds (~20%), we compared the MS/HRMS-based results with those obtained with: (i) aggressive dereplication based on extracted ion chromatograms and isotope patterns, using a search list of all metabolites known from *Penicillium* [20]; and (ii) an unbiased approach based on the Agilent Molecular Feature Extraction (MFE) algorithm which finds all chromatographic peaks and collects adduct, dimeric, and trimeric ions into one feature [62]. The peaks and matching candidates that were identified by the aggressive dereplication approach were evaluated by manually assessing the fragmentation pattern and by using the MassHunter Molecular Structure Correlator program which uses a systematic bond disconnection approach [27]. Likewise, the retention time was compared to the calculated LogD [43], and if possible the UV/Vis data evaluated. This further identified the known compounds chrysogesides B (Figure 7), C, D and E (characteristic loss of glucose and other specific fragments) [63] and three preaustinoids (fragmentations not very specific). Xanthoepocin (Figure 7) [64] was identified and verified from the very specific UV/Vis spectrum and MS/HRMS fragmentations. In full scan positive mode only [M + Na]^+^ and [M + H − H_2_O]^+^ were observed.

The aggressive dereplication approach also identified fellutamides and breviones which are expected from the species [55]; this could, however, not be supported by the MS/HRMS. Most false positive results originated from fragments or adducts of other compounds in the extract. Examples of these were: (i) the loss of acetate from the andrastin A in ESI^+^ matching andibenin B; (ii) quinolactacin A producing [2M + Na]^+^ and [2M + H]^+^ ions matching the [M + Na]^+^ and [M + H]^+^ of fellutanine D, respectively. Close inspection of adduct pattern and retention times, however, showed that andibenin B and fellutanine D were false positives. This underlines the importance of the MS/HRMS dimension for improved confidence in dereplication. False positives are eliminated and compounds that are missed because they are not part of the library can still be verified based on the MS/HRMS data. The unbiased minimum free energy (MFE) algorithm did, as expected, find many more peaks (50%–100%) than the two targeted approaches (data not shown); however, all major peaks in the chromatograms were detected by the targeted approaches, and all major biological activities could be accounted for by compounds in the MS/HRMS library.

#### 2.2.2. Ophiobolins from a Marine-Derived *Aspergillus insuetus*

The extract of a *Aspergillus insuetus* strain (IBT 28443) derived from a sea water sample collected near Greenland was found to have activity in an anticancer assay (unpublished assay). The most potent fractions were found to be enriched in compounds belonging to the ophiobolin family. They are fungal sesterterpenoids with more than 35 known, closely related analogues [1,65]. Of these analogues, eight were available as standards and included in the library. The ophiobolins are known to exhibit a broad spectrum of bioactivities including antifungal and anticancer [1,65].

**Figure 8:**
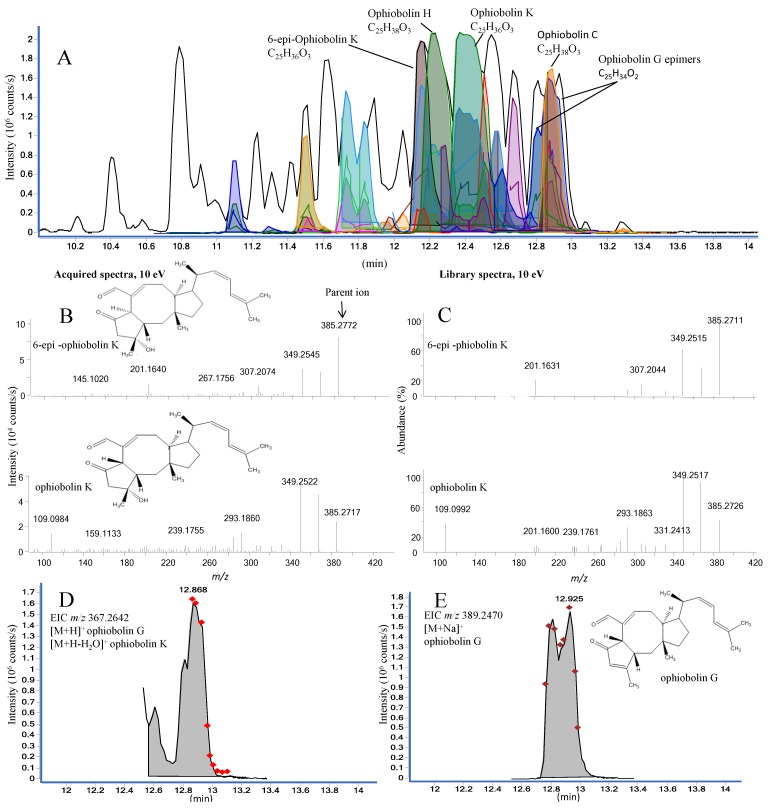
(**A**) Active fraction enriched with compounds from the ophiobolin family in positive ESI mode. More than one color shading of the same peak is either due to different EIC and ECC for same match but different adducts or for other matches that had scored less; (**B**) MS/HRMS acquired spectra (10 eV) for library match of 6-epi-ophiobolin K and ophiobolin K; (**C**) MS/HRMS library spectra (10 eV) of 6-epi-ophiobolin K and ophiobolin K; (**D**) The EIC for the parent *m*/*z* 367.2642 with ophiobolin K as the best library match; (**E**) The EIC for the parent *m*/*z* 389.2470 with ophiobolin G as the library match. The diamond markers indicate number of scans across the peak.

The analysis of a potent fraction is seen in Figure 8A, depicting MS/HRMS library-identified ophiobolins. The identification of four ophiobolins, namely 6-epi-ophiobolin K, ophiobolin H, ophiobolin K, and ophiobolin C, was further corroborated by matching HRMS, retention time, and UV/Vis. Several unidentified ophiobolin analogues seemed to be present in the fraction based on the HRMS and MS/HRMS data.

To illustrate the value of the MS/HRMS library approach, the MassHunter scoring and matching results for the two epimers, 6-epi-ophiobolin K and ophiobolin K (reference standards included in the LC-MS sequence) were compared to demonstrate if compounds varying at only one stereocenter would be unambiguously assigned by the library search. Both reverse and especially forward scoring showed that the epimers could be differentiated based on the intensity for the fragment ions as illustrated for 10 eV in Figure 8 B and C. The forward score for the MS/HRMS of the [M + H]^+^ ion for the 6-epi-ophiobolin K peak was 71% 6-epi-ophiobolin K and 52% ophiobolin K, while it was 71% ophiobolin K and 58% 6-epi-ophiobolin K for the ophiobolin K peak.

For closely related analogues like the ophiobolins, the number of scans and the integration by auto MS/MS highly influence the outcome from the algorithm. This can be seen in Figure 8 for the series of overlapping peaks (between 12.8 and 13.0 min). From the EIC of the MS/HRMS scans of the *m*/*z* 367.2642 ion (Figure 8D) four peaks at 12.60, 12.78, 12.87, and 12.94 integrated as one peak and the average spectrum was matched to ophiobolin K (forward 81%) as the best match which is incorrect, while the likely correct match ophiobolin G (forward 53%) was the second best match. The reason for the incorrect match was both (i) poor peak integration mixing spectra from several compounds, and (ii) that the water loss ion of ophiobolin K was included in the library as it loses water in the ion source. Looking at the structures of ophiobolins K and G, it is apparent that ophiobolin K reacts into ophiobolin G losing water and forming a double bond. The subsequent MS/HRMS spectra of [M + H]^+^ ophiobolin G and [M + H − H_2_O]^+^ ophiobolin K will thus be identical.

This underlines the difficulty of differentiation of isomers based on library matches. Fortunately investigating the [M + Na]^+^ ions (EIC shown in Figure 8E) solves the problem and shows the likely ophiobolin G and 6-epi- ophiobolin G peaks at 12.81 and 12.93 min, respectively. Thus it would strengthen the validity of a compound identification if the matches from the different adducts could be combined and forced to include e.g., the match of the [M + Na]^+^ ion.

#### 2.2.3. Helvolic Acid as the Anti-Microbial Compound in a Marine-Derived *Emericellopsis* sp.

*Emericellopsis* sp. strain (IBT 28361), a possibly new species, was isolated from a sea water sample collected off the coast of the Danish island Fanoe. *Emericellopsis* include both terrestrial and marine species with *E. maritima* being associated with the seaweed Fucus (*Phaeophyceae*) [66]. A potent fraction of the crude extract displayed antibacterial activity against methicillin-resistant *Staphylococcus aureus* (MRSA) [67].

**Figure 9:**
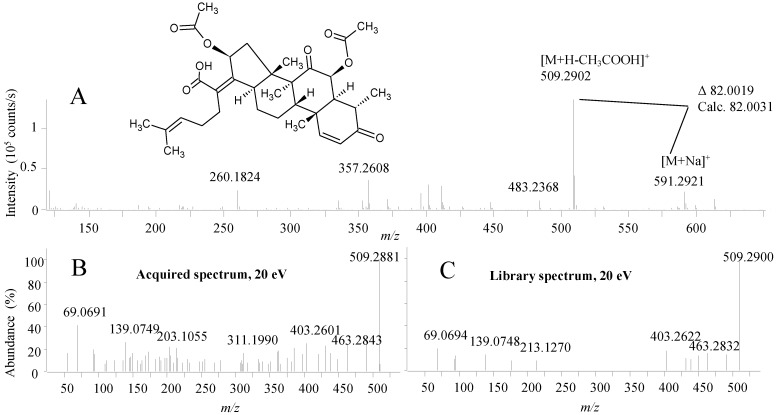
(**A**) The structure of helvolic acid and the ESI^+^ MS spectrum; (**B**) MS/HRMS spectrum at 20 eV from ESI^+^ of helvolic acid and (**C**) the corresponding library spectrum (parent ion *m*/*z* 509).

*Emericellopsis* has not been extensively studied for its chemical potential and thus is it likely that it is not well represented by the compounds in the MS/HRMS library. This was the reason for the very few peaks identified by the MS/HRMS approach compared to the previous cases. Nonetheless, the known antibacterial nortriterpenoid, helvolic acid was identified by MS/HRMS (Figure 9) [68,69] consistent with the biological activity of the fraction. In full scan, ESI^+^ identification was not based on the [M + H]^+^ but rather the accuracy of the fragment at *m*/*z* 509.2902 [C_31_H_41_O_6_]^+^ which was the most abundant peak in the spectrum (Figure 9A). This fragment corresponds to the loss of an O-acetyl group that can be easily lost from the structure of helvolic acid which was verified by the presence of the [M + Na]^+^ at *m*/*z* 591.2921 ion also showing that it was not a deacetyl-helvolic acid. As for the ophiobolins, automated use of both the MS/HRMS spectrum and [M + Na]^+^ from full scan would increase validity of spectral matches. Helvolic acid has formerly been found in related fungal species such as *Emericellopsis terricola* [70] and *Sarocladium oryzae* [71].

The aggressive dereplication approach based on compounds known from *Emericellopsis* and five related genera (*Acremonium*, *Verticillium*, *Chaetomium*, *Sarocladium*, and *Cephalosporium*) likewise only returned few candidates. The total number of annotated peaks was roughly similar for the two methods. Helvolic acid was annotated by both methods, but apart from that there was almost no overlap between the candidates suggested, underlining the inherent bias of the compound library. The MS/HRMS library is biased by mainly containing metabolites from *Penicillium* and *Aspergillus*, while the targeted is generally biased towards the compounds from the most examined genera. The aggressive dereplication matched the anti-protozoal compound, antiamebin I [72], which was originally not included in the MS/HRMS library. However, this compound could be verified later from a reference standard added to the MS/HRMS library. Using the MFE, a series of another five peptaibiotics in the same mass range as antiamebin was detected. These were not detected by the aggressive dereplication approach, as they were not indexed in AntiMarin 2012. However, searching the monoisotopic masses in The Comprehensive Peptaibiotics Database [73] tentatively identified them as different antiamebins (XIII, XIV, XV, III/IV/IX/VII/VIII, and XVI).

## 3. Experimental Section

### 3.1. Strains and Cultivation

All fungal strains used were from the IBT culture collection at the Department of Systems Biology, DTU. The strains described here were *Penicillium antarticum* (IBT 20733 and IBT 27985), *Penicillium bialowiezense* (IBT 28294), *Aspergillus insuetus* (IBT 28443) and *Emericellopsis* sp. (IBT 28361). The marine-derived fungi were cultivated on Czapek yeast extract agar (CYA) and Yeast extract sucrose agar (YES) media for 9 days in the dark at 25 °C [43].

### 3.2. Sample Preparation

Eight plates in total (four CYA and four YES) were extracted with 150 mL ethyl acetate containing 1% formic acid. The crude extracts were fractionated on a reversed phase C_18_ flash column (Sepra ZT, Isolute, 10 g) using an Isolera One automated flash system (Biotage, Uppsala, Sweden). The gradient used was 15%–100% acetonitrile buffered with 20 mM formic acid over 28 min (12 mL/min). Fractions were automatically collected based on UV signal (210 nm and 254 nm). A total of 126 crude, fractions, and blanks were submitted for bioassays antifungal (*A. fumigatus*, *C. albicans*) [74,75], and antibacterial MRSA [67], anticancer (unpublished assay), CNS in zebra fish larvae [51], and anti-inflammatory activity [49].

### 3.3. Standard Metabolites

Secondary metabolite standards have been collected over the past 30 years, either from commercial sources, as gifts from other research groups, or purified from our own projects [43,76], hence their quantity and purity varies (micro- to milligram quantity, ≥50% purity). The collection contains approximately 1600 standards with 95% of them being of fungal origin (5% of bacterial origin). Commercial sources of purchased standards include Sigma-Aldrich (Steinheim, Germany), Axxora (Bingham, UK), Cayman (Ann Arbor, MI, USA), TebuBio (Le-Perray-en-Yvelines, France), Biopure (Tulln, Austria), Calbiochem (San Diego, CA, USA), ICN (Irvine, CA, USA), Bachem GmbH (Weil am Rhein, Germany), and AnalytiCon Discovery GmbH (Potsdam, Germany). All standards were kept dry at −20 °C and, unless stated otherwise, were dissolved in 140 µL acetonitrile prior to analysis. If not soluble in pure acetonitrile, 50% acetonitrile in MilliQ water was used. Prepared standard solutions were also preserved on −20 °C.

### 3.4. UHPLC-DAD-QTOFMS Analysis

Ultra-high performance liquid chromatography-diode array detection-quadruple time of flight mass spectrometry (UHPLC-DAD-QTOFMS) was performed on an Agilent Infinity 1290 UHPLC system (Agilent Technologies, Santa Clara, CA, USA) equipped with a diode array detector. Separation was obtained on an Agilent Poroshell 120 phenyl-hexyl column (2.1 × 150 mm, 2.7 µm) with a linear gradient consisting of water (A) and acetonitrile (B) both buffered with 20 mM formic acid, starting at 10% B and increased to 100% in 15 min where it was held for 2 min, returned to 10% in 0.1 min and keeping it for 3 min (0.35 mL/min, 60 °C). Injection volume, depending on sample concentration, typically varied between 0.1 and 1 µL. To avoid carry-over, the auto-sampler was operated in the flow-through-needle mode and further coupled to an Agilent Flex Cube which was used to back flush the needle seat for 15 s. at a flow of 4 mL/min with each of: (i) isopropanol: 0.2% ammonium hydroxide in water (1:1 *v/v*); (ii) acetonitrile with 2% formic acid; (iii) water with 2% formic acid.

MS detection was done on an Agilent 6550 iFunnel QTOF MS equipped with Agilent Dual Jet Stream electrospray ion source with the drying gas temperature of 160 °C and gas flow of 13 L/min and sheath gas temperature of 300 °C and flow of 16 L/min. Capillary voltage was set to 4000 V and nozzle voltage to 500 V. Ion-source parameters were the same for ESI^+^ and ESI^−^ mode. Mass spectra were recorded as centroid data for *m*/*z* 85–1700 in MS mode and *m*/*z* 30–1700 in MS/MS mode, with an acquisition rate of 10 spectra/s. Automated data-dependent acquisition MS/HRMS (auto-MS/HRMS) analysis was commonly done for ions detected in the full scan above 50,000 counts (may be adjusted for low/high concentration samples) with a cycle time of 0.5 s, the quadrupole isolation width in narrow (*m*/*z* ±0.65), using fixed CID energies of 10, 20, and 40 eV and maximum three selected precursor ions per cycle. A narrow exclusion time of 0.04 min was used to get MS/MS of less abundant ions when compounds co-eluted.

Lock mass solution in 95% acetonitrile was infused in the second sprayer using an extra LC pump at a flow of 10–50 µL/min, the solution contained 1 µM tributyle amine (Sigma-Aldrich), 10 µM Hexakis(2,2,3,3-tetrafluoropropoxy)phosphazene (Apollo Scientific Ltd., Cheshire, UK), and 1 µM trifluoroacetic acid (Sigma-Aldrich) as lock masses. The [M + H]^+^ ions of first two (*m*/*z* 186.2216 and 922.0098 respectively) were used in positive mode, while [M + HCOO]^−^ and [M − H]^−^ of the latter two were used in negative mode (*m*/*z* 966.0007 and 112.9856). 

### 3.5. Library Setup and Auto-MS/MS Data Analysis

The MS/HRMS library was constructed from our internal ChemFolder library (Advanced Chemical Developments, Toronto, ON, Canada) of 7400 compounds of which 1600 were available as reference standards [20]. For reference standards and tentatively identified compounds, name, structure, and CAS no. were transferred to the Agilent Masshunter PCDL manager 4.00 (Service release 1), and linked to the retention time and MS/HRMS spectra of 10, 20, and 40 eV, either by manually pasting from MassHunter or imported via a cef file. All major pseudomolecular ions ([M + H]^+^, [M + Na]^+^, [M + NH_4_]^+^, [M − H]^−^, [M + HCOO]^−^), and simple fragment ions (mainly [M + H − (H_2_O)*_n_*]^+^, [M + H − CH_3_COOH]^+^, [M – H − CO_2_]^−^) which provided characteristic MS/MS spectra were included. 

Data files were processed by the Find by Auto MS/MS function in Masshunter, usually without any intensity threshold but often with a limit to the 200 largest peaks, mass match tolerance *m*/*z* 0.05. Unless otherwise stated the MS/HRMS library was searched using precursor and product ion expansion of 50 ppm + 2 mDa as well as minimal reverse and forward scores of 50 each.

### 3.6. Aggressive Dereplication and Molecular Feature Extraction

For analysis of compounds described in the literature and not necessarily available as reference standards, Aggressive dereplication (Klitgaard *et al.* 2014 [20]) was performed on the ESI^+^ and ESI^−^ full scan data using the *Find by Formulae* function in Agilent Masshunter Qualitative analysis B06.00 software. The following adducts and common fragments were included: ESI^+^, [M + H]^+^ and [M + Na]^+^; ESI^−^, [M − H]^−^, [M + HCOO]^−^. All ions analyzed were treated as being singularly charged. The area cut-off was set to 10,000, and the mass spectrum was recorded below 10% of the height of the peak to avoid detector overload. A minimum score of 70 was used to ensure that only compounds with fitting isotope patterns were annotated. 

The search lists were constructed from the AntiMarin2012 which was converted into an sdf-database and then imported into ChemFolder and from here to Excel (Klitgaard *et al.* 2014 [20]) where it was formatted to the Agilent search list format. All this work was made on an AntiMarin-licensed computer.

The MFE screening was performed in the Agilent Masshunter Qualitative analysis B06.00. The following adducts and common fragments were included: ESI^+^, [M + H]^+^ and [M + Na]^+^; ESI^−^, [M − H]^−^, [M + HCOO]^−^. All ions analyzed were treated as being singly charged. The area cut-off was set to 10,000, and the mass spectrum was recorded below 10% of the height of the peak to avoid detector overload. A minimum quality score of 99 was used to ensure that only compounds with fitting mass, isotope patterns, and peak shape were annotated.

## 4. Conclusions

In this work we demonstrate that MS/HRMS search in a library is a robust and reliable way of tentatively identifying known bioactive compounds on a single instrument. With spectra reproducibility across Agilent instruments [26,77] the library should be directly usable on these, while others instruments presumably need adjustment against collision energies (e.g., 10 eV on the Agilent may correspond to 15 eV on a Bruker QTOF). Furthermore MS/HRMS aided the tentative identification of novel isomers, e.g., to be used in bioactivity optimization. Many highly bioactive compounds are found across the fungal kingdom, and even when exploring specialized marine environments where it is likely to find novel bioactive compounds it is of outmost importance to identify known nuisance compounds in the first screen. To aid drug discovery dereplication we thus suggest that it is required to deposition MS/MS spectra of all novel published compounds in Massbank, MetLin and/or GNPS [31], although for all mentioned an easy interface for depositing spectra is needed. 

Aggressive dereplication of full scan data supplemented by auto MS/HRMS to strengthen the correct match and elimination of false positives proved efficient and could in many cases be strengthened even further by UV/Vis data. 

Both described strategies can handle extracts produced months in-between which is a problem for the unbiased peak picking and adduct pattern algorithms which in general requires samples to be run within a sequence and with replicated and blank samples to handle variations in chromatographic separation, mass spectra, sample preparation, and growth media. Nonetheless, an unbiased peak picking strategy was the only way to detect a series of non-data based compounds as demonstrated in the last case, proving the need to integrate many data-analysis strategies and tools to obtain comprehensive compound coverage.
